# Variations in Transporter Genes are Linked to Region-Specific Patterns of Cortical Brain Aging

**DOI:** 10.1093/geroni/igaf122.3682

**Published:** 2025-12-31

**Authors:** Nicholas Kim, Ayati Mishra, Nahian Chowdhury, Andrei Irimia

**Affiliations:** University of Southern California, Los Angeles, California, United States; University of Southern California, Los Angeles, California, United States; University of Southern California, Los Angeles, California, United States; University of Southern California, Los Angeles, California, United States

## Abstract

Deep learning-derived local brain age (LBA) provides a regional indicator of cortical aging, with higher values reflecting more senesced brain regions. Elucidating the genetic architecture of LBA can reveal mechanisms underlying individual differences in brain aging and susceptibility to neurodegeneration. We conducted the first genome-wide association study of LBA in 41,708 cognitively normal adults (21,983 females) aged 45 to 83 years in the UK Biobank, testing genetic variants for associations with regional cortical aging. Our analysis identified 1,212 significant variants, with the most prominent signals mapping to genes involved in ion and metabolite transport. Two independent variants near KCNK2, which encodes a potassium channel, exhibited the strongest associations with LBA. These alleles were linked to faster aging in the cingulate, precuneus, and parietal cortices, brain regions that undergo early atrophy in Alzheimer’s disease (AD). Additional associations implicated variants mapped to SLC25A13, a mitochondrial aspartate-glutamate carrier, and SLC6A20, a transporter of proline and glycine. The SLC25A13 variant was associated with slower aging in frontal and temporal cortices. These brain regions support executive function and episodic memory, both disrupted in AD. In contrast, the SLC6A20 variant was linked to faster aging across parietal and sensorimotor cortices, regions implicated in neurodegenerative motor symptoms such as gait disturbance in Parkinson’s disease. Together, these findings suggest a polygenic architecture of cortical aging anchored in transport pathways, highlighting potential targets for early intervention. Future work will test if polygenic risk scores capturing the combined effects of transport-related variants can predict trajectories of regional cortical aging.

